# Epigenetic Regulation of Subgenomic Gene Expression in Allotetraploid *Brassica napus*

**DOI:** 10.3390/plants12142608

**Published:** 2023-07-10

**Authors:** Meimei Hu, Zengde Xi, Jianbo Wang

**Affiliations:** State Key Laboratory of Hybrid Rice, College of Life Sciences, Wuhan University, Wuhan 430072, China; meimeihu@whu.edu.cn (M.H.); xizengde@whu.edu.cn (Z.X.)

**Keywords:** allotetrapolyploid, *Brassica napus*, subgenome, gene expression, epigenetics, DNA methylation, histone modification

## Abstract

The allotetraploid *Brasscia napus* has now been extensively utilized to reveal the genetic processes involved in hybridization and polyploidization. Here, transcriptome, WGBS, and Chip-Seq sequencing data were obtained to explore the regulatory consequences of DNA methylation and histone modifications on gene expression in *B. napus*. When compared with diploid parents, the expression levels of 14,266 (about 32%) and 17,054 (about 30%) genes were altered in the A_n_ and C_n_ subgenomes, respectively, and a total of 4982 DEGs were identified in *B. napus*. Genes with high or no expression in diploid parents often shifted to medium or low expression in *B. napus*. The number of genes with elevated methylation levels in gene promoters and gene body regions has increased in A_n_ and C_n_ subgenomes. The peak number of H3K4me3 modification increased, while the peak number of H3K27ac and H3K27me3 decreased in A_n_ and C_n_ subgenomes, and more genes that maintained parental histone modifications were identified in C_n_ subgenome. The differential multiples of DEGs in *B. napus* were positively correlated with DNA methylation levels in promoters and the gene body, and the differential multiples of these DEGs were also affected by the degree of variation in DNA methylation levels. Further analysis revealed that about 99% of DEGs were of DNA methylation, and about 68% of DEGs were modified by at least two types of DNA methylation and H3K4me3, H3K27ac, and H3K27me3 histone modifications. These results demonstrate that DNA methylation is crucial for gene expression regulation, and different epigenetic modifications have an essential function in regulating the differential expression of genes in *B. napus*.

## 1. Introduction

Epigenetics refers to events that alter phenotype, morphology, or molecular structure through various regulatory mechanisms without involving changes in DNA sequence, which are prevalent during interspecific hybridization and polyploidization [1]. DNA methylation is an important part of epigenetics, which can inhibit gene expression, but relevant studies have shown that DNA methylation mediated by SUVH1 and SUVH3 can promote gene expression [2,3]. DNA methylation modification determines the accessibility of genetic material, and dynamic regulation of specific DNA methylation includes the establishment, maintenance, and activity clearance (i.e., demethylation) of DNA methylation [4]. Histone modification includes acetylation, methylation, ubiquitination, phosphorylation, glycosylation, and other modifications. As one of the most complex epigenetic modifications, histone modification can be methylated at different sites of lysine and arginine and is closely related to DNA methylation, chromatin structure and gene activity [5]. Histone methylation and acetylation modifications can be established and removed by corresponding enzymes. H3K9ac and H3K4me3 are commonly found in euchromatin and associated with active transcription, while H3K9me2 and H3K27me3 are commonly found in heterochromatin and associated with gene suppression [6].

Polyploidy is a universal feature of plant genomes, and polyploids are common in angiosperms [7]. Polyploids have stronger adaptability, and the phenotypic changes caused by polyploidy are of great value for crop breeding. Epigenetic changes contribute to the genetic diversity and diploidization of allopolyploids, and they are essential for the genome stabilization and evolution of polyploids [8]. Synthetic and natural *Arabidopsis* allotetraploids have shown that genomic balance is accompanied by coordinated changes in DNA methylation between subgenomes after allopolyploidization [9]. Dynamic and reversible changes in chromatin and DNA methylation caused by genomic merging and separation are closely related to changes in gene expression and transposon activity [10]. Allotetraploid cotton showed a biased H3K4me3 histone modification level [11]. Salt stress in tetraploid rice has shown that polyploidization induces hypomethylation of CHH, which enables rapid responses to environmental stimuli and, thus, a rapid response to salt stress [12]. In a study of *Arabidopsis*, loss of DNA methylation modification resulted in a loss and redistribution of histone modification [13]. H3K27me2 modification in wheat plays an important role in inhibiting CATA transposons produced by polyploidy and preventing plant genetic recombination [14]. The inhibition of DNA methylation on gene expression and transposable elements was highly dose-dependent [15]. DNA hypomethylation induced by polyploidy is important for improving the fitness of polyploid plants [12].

*Brassica* plants are widely cultivated as vegetables and oil crops, which have brought great economic value for human development. Several representative species of *Brassica* can be described by the U’s Triangle, including three diploid species, *B. rapa* (AA), *B. nigra* (BB), and *B. oleracea* (CC), and three tetraploid species, *B. juncea* (AABB), *B. napus* (AACC), and *B. carinata* (BBCC) [16]. The whole-genome sequencing results of *Brassica* plants such as *B. rapa* and *B. oleracea* showed that *Brassica* experienced additional WGT (whole-genome triploidization) events compared with Arabidopsis, which played an important role in the morphological diversity of *Brassica* species [17]. A large number of newly synthesized *B. napus* showed substantial aneuploidy [18], and there may be a shared pattern of karyotype evolution in Brassicaceae after polyploidy [7]. Studies have revealed the phenomenon of asymmetric genomic expression in polyploids [19]. As an allopolyploid, the two subgenomes of *B. napus* experienced bias separation after polyploidy, resulting in asymmetric genome evolution [20]. There are many complex factors affecting polyploid evolution, such as the asymmetric loss of subgenomes, the asymmetric amplification of TEs and tandem repeats, and differences in DNA sequence and expression [19]. Bias segregation resulted in varying degrees of gene loss [21], and the polyploidy and loss processes resulted in changes in the number of gene families, which may contribute to the morphological plasticity of *Brassica* species [16]. The A subgenome in *B. napus* and *B. juncea* exhibits a higher frequency of genetic recombination and maintains a higher nucleotide diversity than the C or B subgenome, but the mechanism for this remains unclear [22]. Differences in ecological, physiological, morphological, and genetic traits after allopolyploidy cannot be simply attributed to hybridization or WGD (whole-genome duplication) processes, and they are considered a comprehensive effect of hybridization, polyploidy, and subsequent evolutionary processes [7]. The synthesis of new allotetraploids is of great significance for revealing the changes that polyploids naturally undergo during polyploidization and subsequent evolution, and the effects of hybridization, genome duplication, and subsequent evolution can be distinguished by comparison with diploid parents. At present, the generation of polyploids by resynthesis has been widely used in genome research [9,10,23,24,25].

Although the characteristics of *B. napus* gene expression and epigenetic modification have been studied [26], these analyses mainly concentrated on homologous genes, and the epigenetic factors which impact the DEGs at the early stage of *B. napus* formation have not been investigated. In this study, a resynthetic *B. napus* and its diploid parents were the research materials, and the genome of *B. napus* acted as the common reference genome to examine the gene expression and epigenetic characteristics among the subgenomes of *B. napus*. Additionally, the epigenetic factors that regulate the differential expression of genes were explored. The consequences can provide a reference for comprehending the allopolyploid epigenetic mechanism.

## 2. Results

### 2.1. Subgenomic Gene Expression in B. napus

**Figure 1:**
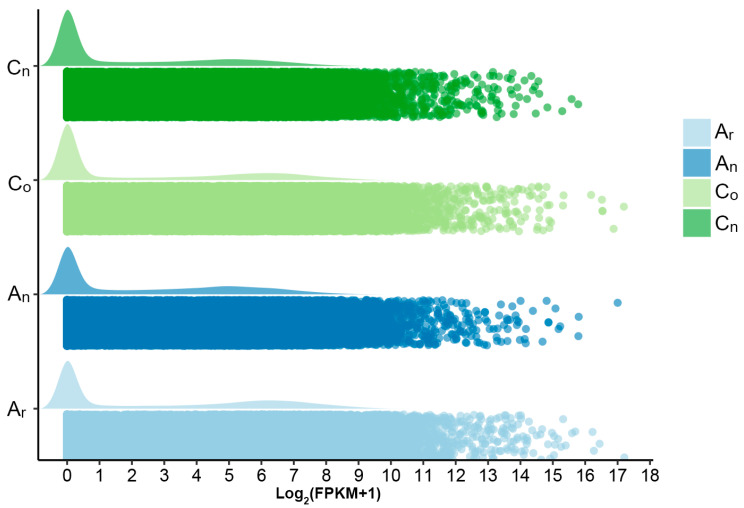
Distribution of *B. rapa* (A_r_), *B. oleracea* (C_o_), and *B. napus* whole-genome expression levels. A_r_, C_o_, A_n_ and C_n_ represent *B. rapa*, *B. oleracea,* and the A_n_ and C_n_ subgenomes of *B. napus*, respectively.

Variations in gene expression throughout the whole genome can reveal changes in expression levels in *B. napus*. The distribution of expression data in Figure 1 shows that the A_n_ and C_n_ subgenomes of *B. napus* had the same number of genes as the A_r_ and C_o_ subgenomes of *B. rapa* and *B. oleracea*, respectively. However, as compared to that of the diploid parents, the number of genes with high expression levels was reduced and the number of genes with medium expression levels was increased in the two subgenomes of *B. napus*. It was hypothesized that genes with intermediate expression levels were more prevalent in *B. napus* than in the diploid parents.

**Figure 2:**
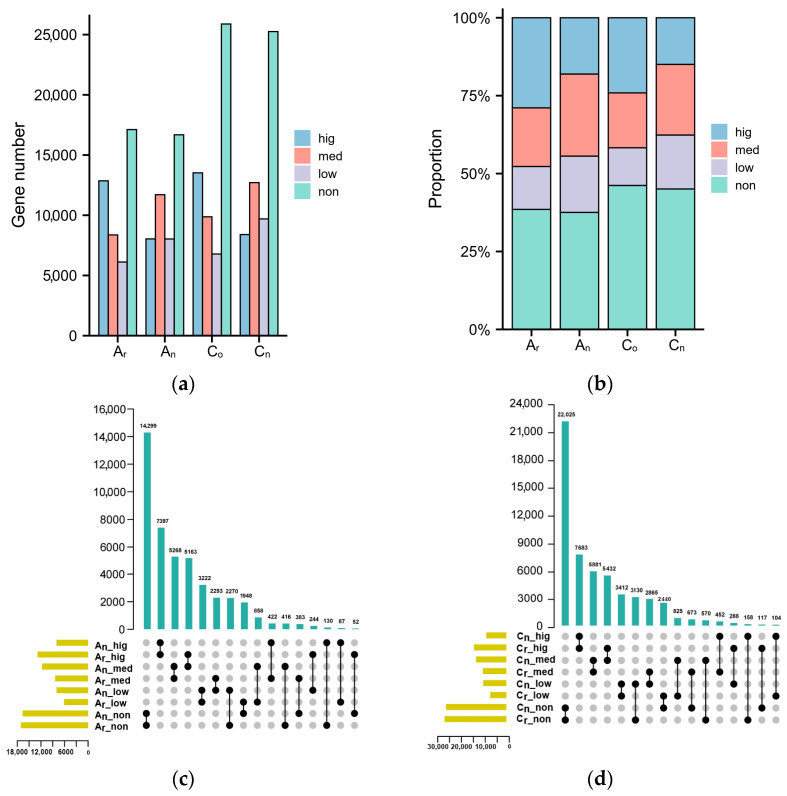
Analysis of gene expression level variation in *B. napus* and its diploid parents. (**a**) The number of genes at different expression regions of *B. rapa*, *B. oleracea*, and *B. napus*. (**b**) The proportion of genes with different expression levels in *B. napus* and its diploid parents. (**c**) Genes in *B. rapa* and the A_n_ subgenome of *B. napus* overlap at different expression levels. (**d**) Genes in the C_n_ subgenome of *B. napus* and *B. olerecea* overlap at all expression levels. No expression, low expression, medium expression, and high expression are denoted by the letters non, low, med, and hig, respectively.

Gene expression levels were classified into four categories based on their FPKM values: no expression (FPKM < 0.001), low expression (0.001 ≤ FPKM < 5), medium expression (5 ≤ FPKM < 50), and high expression (FPKM > 50). In comparison to diploid parents, the number and proportion of genes expressed at low and medium expression levels increased, but the number and proportion of genes with high and no expression levels declined in *B. napus* (Figure 2a,b). *B. napus* and its diploid parents underwent an overlapping analysis of gene expression (Figure 2c,d). The findings demonstrated that most genes (30,186, nearly 68%) in the A_n_ subgenome kept their original expression level. About 32% (14,266) of genes changed their expression levels, with 5163 genes switching from high expression in the parents to medium expression in the offspring, 2293 genes alternating from moderate expression to low expression, 2270 genes converting from no expression to low expression, and 1948 genes changing from low expression to no expression. In the C_n_ subgenome, the expression levels of 39,001 (about 70%) genes were unaltered, whereas 17,054 (almost 30%) genes underwent changes. In terms of the genes whose expression levels changed, 5432 genes changed from being highly expressed in the parents to being moderately expressed in the offspring, 3130 genes changed from no expression to low expression, 2865 genes shifted from medium expression to low expression, and 2440 genes changed from low expression to no expression.

In contrast to the diploid parents, more gene expression levels were altered in the C_n_ subgenome of *B. napus*. The majority of the genes with expression changes in the A_n_ and C_n_ subgenomes changed from high to medium expression, and many genes altered from medium to low expression. According to these results, most genes were expressed at lower levels in *B. napus* than in its diploid parents.

### 2.2. Gene Expression Levels Verified by Real-Time Quantitative PCR (RT-qPCR)

**Figure 3:**
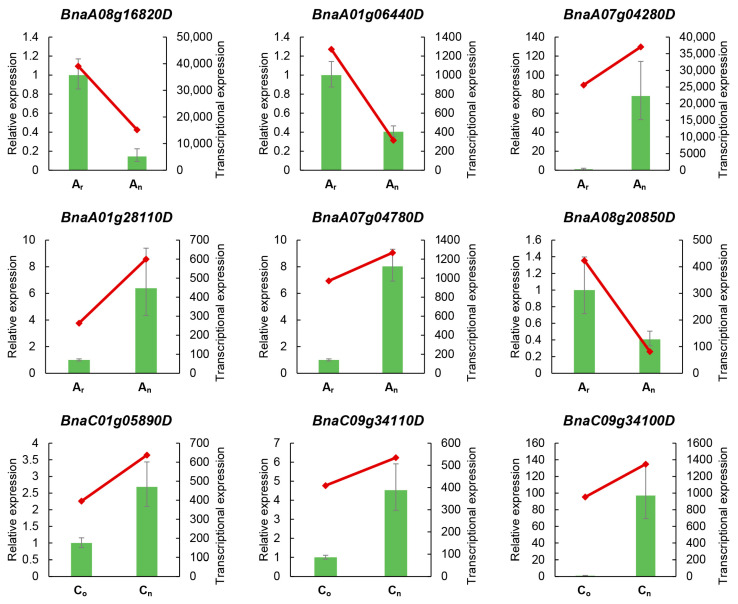
The RT-qPCR and transcriptome sequencing results of 9 genes. Histogram represents expression data from RT-qPCR, and line chart indicates expression levels obtained by transcriptome sequencing. The left ordinate represents the expression level obtained from RT-qPCR experiments. The right ordinate represents the expression level of transcriptome sequencing.

RT-qPCR is a common technique to detect gene expression during a research process. In this instance, 9 genes from *B. napus* were randomly chosen for RT-qPCR (Figure 3). Among the 9 genes, 3 genes were expressed at a lower level in *B. napus* than in its diploid parents, and 6 genes were expressed at a lower level in the diploid parents than in *B. napus*. All the gene expression levels verified by RT-qPCR in *B. napus* and its diploid parents were consistent with transcriptome sequencing results, showing the reliability of the high-throughput sequencing-obtained expression levels.

### 2.3. Detection and Analysis of Methylation Sites

**Figure 4:**
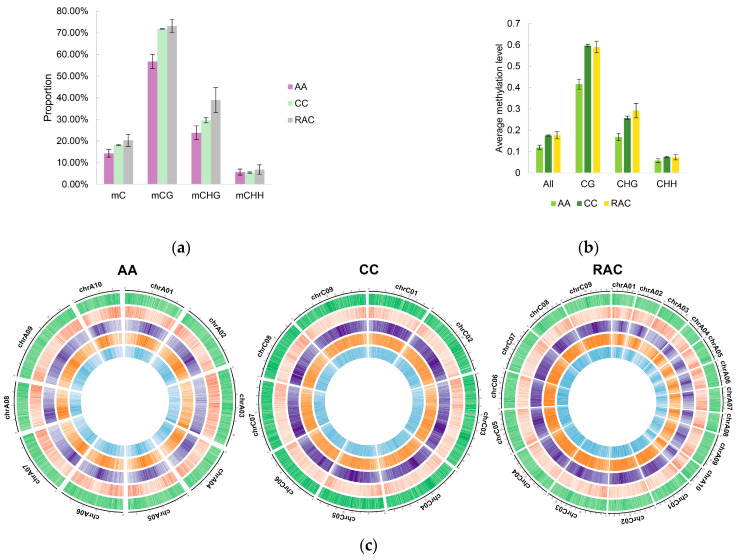
Methylation sites in *B. napus* and its diploid parents have been identified and analyzed. (**a**) The percentage of methylated cytosine in *B. rapa* (AA), *B. oleracea* (CC), and *B. napus* (RAC). (**b**) Average CG, CHG, and CHH methylation levels in *B. napus* and its diploid parents. (**c**) Cisco map of methylation levels in *B. napus* and its diploid parents. GC content (green), gene density (red), CG methylation level (purple), CHG methylation level (orange), and CHH methylation level (light blue) are displayed sequentially from the outside in. The level or density is greater the darker the color is. The ordinate of Figure A indicates the proportion of methylated cytosine (e.g., mCG proportion = mCG/CG). Figure B shows the average methylation level of the whole genome.

In *B. napus*, the proportion of methylated cytosine grew, and the relative amounts of mCG, mCHG, and mCHH (where H stands for A, T, and C) also increased to varied degrees, with mCHG increasing the most (Figure 4a). *B. napus* had a much greater whole methylation level than *B. rapa*, although it was only marginally higher than *B. oleracea*. The average methylation levels of CG and CHH were also greater than *B. rapa* and somewhat below *B. oleracea* (Figure 4b), whereas the average methylation level of CHG was higher than that of diploid parents in *B. napus*. The distribution of the methylation levels of CG, CHG, and CHH in *B. napus* was more similar to that in *B. oleracea*, as shown by the Cisco diagram (Figure 4c).

**Figure 5:**
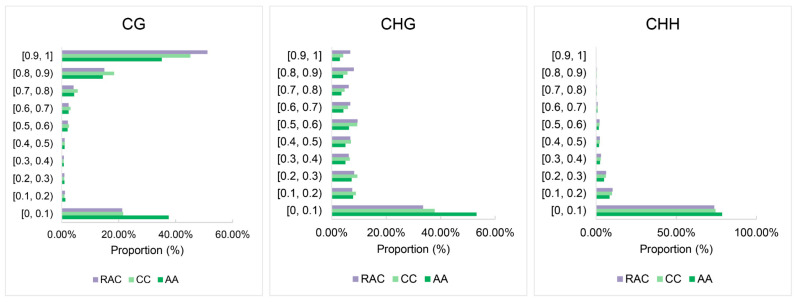
Distribution of methylation level at the C site in the diploid parents and *B. napus*. The ordinate represents the range of methylation level distribution. “[” indicates that the left value is included, while “)” indicates the right value is not included; for example, [0, 0.1) shows the methylation level is more than or equal to 0 and less than 0.1 (this is also the case in the following figures).

The methylation levels of the CG methylation sites in the three species were primarily distributed in the high methylation range of 0.9 to 1, whereas the methylation levels of the CHG and CHH methylation sites were mostly distributed in the low methylation range of 0 to 0.1, according to the results of the assessment of the distribution of cytosine methylation levels (Figure 5). *B. napus* showed a rise in the percentage of methylated sites with high methylation levels when compared to its diploid parents, particularly for CG and CHG methylation. *B. napus* has more genes identified in the high methylation level intervals than its parents, whereas fewer genes were found in the low methylation level intervals.

### 2.4. Changes of Subgenomic DNA Methylation in B. napus

**Figure 6:**
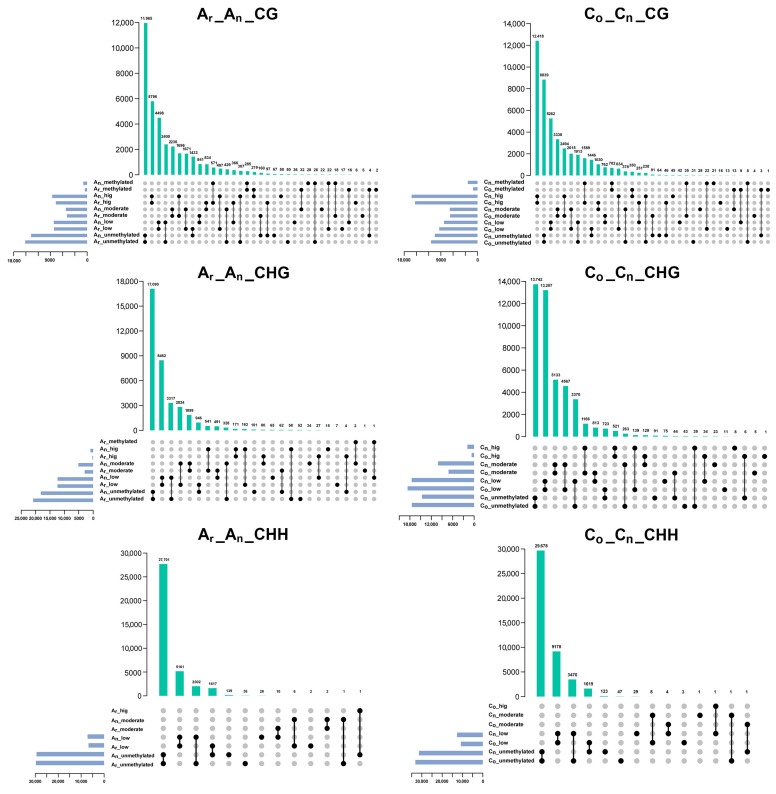
Analysis of methylation level changes in *B. napus* promoter regions. The titles on each figure reflect changes in different methylation levels in the A_n_ and C_n_ subgenomes of *B. napus* and its diploid parents. For instance, A_r__A_n__CG denotes the alteration in the level of CG methylation in the A_n_ subgenome of *B. napus* compared to *B. rapa*, and so on (similarly in the next figure).

**Figure 7:**
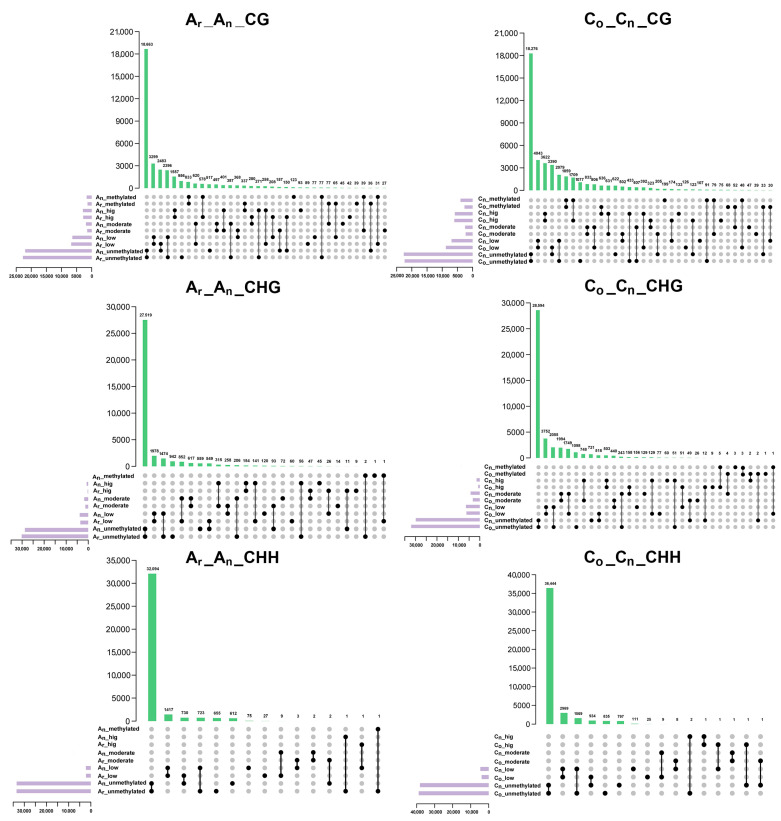
Analysis of methylation level changes in *B. napus* gene body regions.

According to the distribution of methylation levels in the five intervals of [0, 0.1), [0.1, 0.4), [0.4, 0.6), [0.6, 0.9), and [0.9, 1), the methylation levels were divided into unmethylated, low methylated, moderately methylated, high methylated, and fully methylated. The methylation of the promoter (Figure 6) and gene body (Figure 7) overlapped in *B. napus* and its diploid parents. In the figure, unmethylated, low, moderate, high, and methylated denote various levels of methylation, namely unmethylated, low methylated, moderately methylated, high methylated, and fully methylated.

For promoters, a total of 11,696 genes experienced methylation level changes in the A_n_ subgenome, with 7587 (about 65%) genes displaying an increase in methylation level in the promoter region. The methylation levels of 8844 genes in CHG methylation were altered, in which 7231 genes (82%) were increased. A total of 3637 genes changed their promoter CHH methylation levels, with 2009 genes (about 55%) exhibiting elevating methylation. In conclusion, regardless of the type of methylation, most gene promoter regions in the A_n_ subgenome exhibited raised methylation levels.

In the C_n_ subgenome, 13,321 gene promoter regions had altered CG methylation, with 9320 (about 70%) exhibiting increased methylation. A total of 11,293 genes’ GHG promoter methylation levels revealed alterations, and 9505 (nearly 84%) of those genes exhibited greater methylation levels. For promoter CHH methylation, 5104 genes’ methylation levels changed, with 3479 genes (around 68%) displaying increasing methylation levels. Consistent with the A_n_ subgenome, there were more gene promoter regions with higher methylation levels than in *B. oleracea* in the C_n_ subgenome.

In gene body regions (Figure 7), a total of 9219 genes with CG methylation in the A_n_ subgenome demonstrated alterations in methylation levels, of which 5058 (about 55%) genes had elevated methylation levels. A total of 4031 genes revealed changes in the gene body’s CHG methylation, with 3047 genes (about 76%) exhibiting an increased methylation level. The gene body CHH methylation of 1470 genes was altered, of which 734 genes (about 50%) displayed elevated methylation levels. For the C_n_ subgenome, the CG methylation level was altered in 12,143 genes, of which 6675 (about 55%) genes exhibited increased methylation levels. The methylation level of CHG was altered in 6217 genes in total, with 4981 genes (nearly 80%) having higher methylation levels. Moreover, 2525 genes had altered gene body methylation levels in CHH methylation, while 1580 (about 63%) genes displayed increased gene body methylation. These results revealed that the promoter and gene body methylation levels in *B. napus* were elevated compared to that of its diploid parents.

### 2.5. Effects of DNA Methylation on Gene Expression

DNA methylation, a significant epigenetic mechanism, is essential for gene expression regulation. According to the proportion of the distribution of gene numbers, the number of genes in the high expression level dropped as the promoter methylation level rose (Figure S1). For genes that were not expressed, the number of genes rose as the level of promoter methylation increased. The distribution pattern of DNA methylation and gene expression in the gene body region is similar to that in the promoter region (Figure S2). This pattern appeared to be more pronounced in the gene body than in the promoter region, with CG being more pronounced than CHG and CHH.

**Figure 8:**
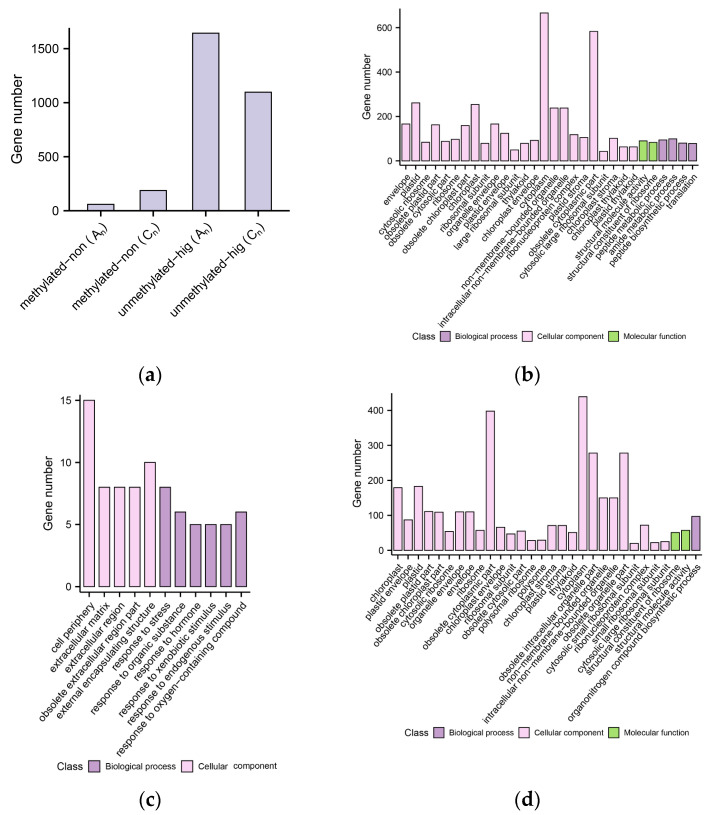
Number and functional annotation of genes in *B. napus* that are conserved in DNA methylation and expression. (**a**) The number of conserved genes whose methylation levels were extremely negatively correlated with gene expression levels in the subgenomes of A_n_ and C_n_. (**b**) The GO annotation terms for genes in a subgenome that are not methylated but are nevertheless highly expressed. (**c**) The GO annotation terms of fully methylated but non-expressed genes in C_n_ subgenome. (**d**) GO annotation terms for the C_n_ subgenome’s non-methylated but highly expressed genes.

In order to further investigate the expression of genes with conserved methylation types and levels in both the gene body and promoter regions in *B. napus*, we carried out identification statistics and GO annotation analysis for these genes (Figure 8). Because there are a few methylated CHG and CHH genes in this study (less than 10 genes), only the genes with fully methylated CG levels and no expression were considered. Furthermore, there were so few completely methylated and unexpressed genes for CG that were found in the A_n_ subgenome that there is no discernible functional enrichment. Therefore, we only analyzed GO enrichment for the other three examples.

In both subgenomes, there are many fewer completely CG-methylated and non-expressed conserved genes than CG-unmethylated and highly expressed conserved genes. The C_n_ subgenome had more complete CG-methylated and unexpressed conserved genes, whereas the A_n_ subgenome contained more CG-unmethylated and highly expressed conserved genes. More genes tended to preserve CG-unmethylated and highly expressed patterns in *B. napus* compared to their diploid parents, and this tendency was more pronounced in the A_n_ subgenome. The functions of non-methylated and highly expressed conserved genes are most annotated in several functional categories associated with cell composition in the A_n_ and C_n_ subgenomes. For completely CG-methylated and non-expressed genes, functional enrichment can only be carried out in C_n_, and most of these genes were annotated as response-related functions. To sum up, more genes that are involved in cell composition functions are conserved in the CG methylation level of the gene body and promoter and maintained similar expression levels in *B. napus* compared to their diploid parents.

### 2.6. Subgenomic Histone Modification in B. napus

**Figure 9:**
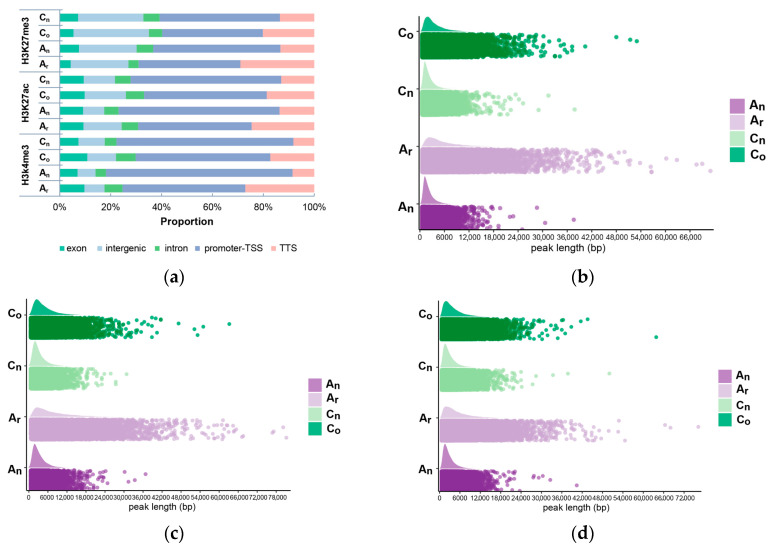
H3K4me3, H3K27ac, and H3K27me3 peak proportions in gene regions and length distribution in *B. napus* and its diploid parents. (**a**) The peak number percentage of the histone modifications in gene regions. (**b**–**d**) H3K4me3 (**b**), H3K27ac (**c**), and H3K27me3 (**d**) peak length distribution.

Histone modification is another significant epigenetic mechanism. Here, we explored the peak counts (Table 1), lengths, and spatial distributions (Figure 9) of the H3K4me3, H3K27ac, and H3K27me3 histone modifications identified in *B. napus* and its diploid parents.

In comparison to the A_r_ and A_n_ subgenomes, the C_o_ and C_n_ subgenomes presented a larger peak number of histone modifications. When compared to the diploid parents, *B. napus* showed a decrease in the peak number of H3K27ac and H3K27me3 modifications and an increase in the peak number of H3K4me3 modifications in the A_n_ and C_n_ subgenomes. Additionally, the proportion of histone modification identified in the promoter to transcription start site regions was elevated, whereas the proportion identified in the transcription end site declined in both the A_n_ and C_n_ subgenomes. The peak length of all observed histone modifications was concentrated in a shorter length range.

**Figure 10:**
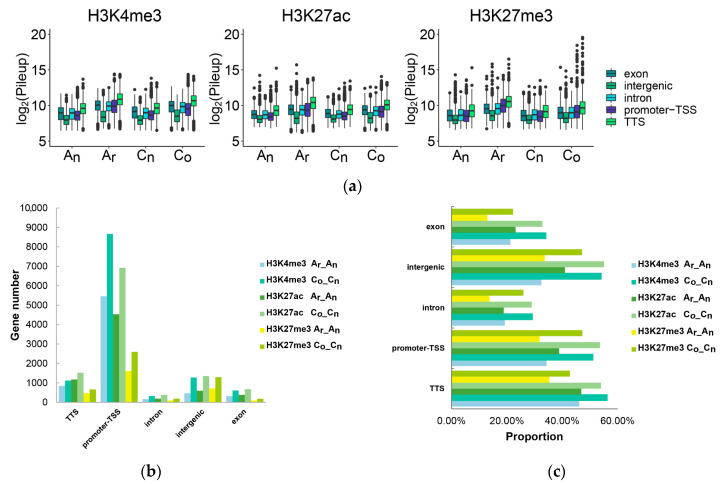
The pileup distribution (**a**), conserved gene number (**b**), and proportion (**c**) of histone modifications in gene regions and intergenic regions of *B. napus* and its diploid parents. A_r__A_n_ indicated that histone modifications occurred in *B. rapa* and the A_n_ subgenome of *B. napus*, and C_o__C_n_ indicated that histone modifications occurred in *B. oleracea* and the C_n_ subgenome of *B. napus* (the proportion in figure c is the number of genes unchanged by histone modification in *B. napus* in this region/the total number of corresponding histone modification genes in corresponding subgenomes in *B. napus* in this region).

Further investigation of the identified histone modification levels revealed that all gene regions in the A_n_ and C_n_ subgenomes of *B. napus* had lower histone modification levels than their diploid parents (Figure 10a). The results revealed that most conserved histone modification peaks were found in the region between the promoter and transcription start site (Figure 10b,c). The fraction of conserved histone modifications in exons and introns was lower than that observed in other regions, while the number and proportion of conserved histone modifications identified in all regions of the C_n_ subgenome were higher than those of the A_n_ subgenome.

### 2.7. The Impact of Histone Modification on Gene Expression

**Figure 11:**
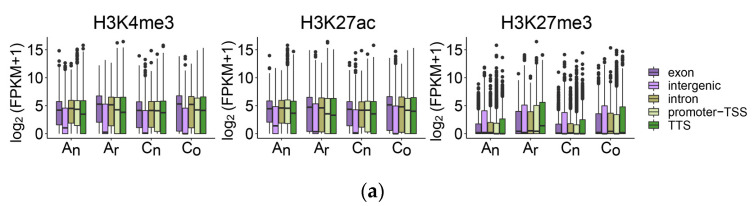

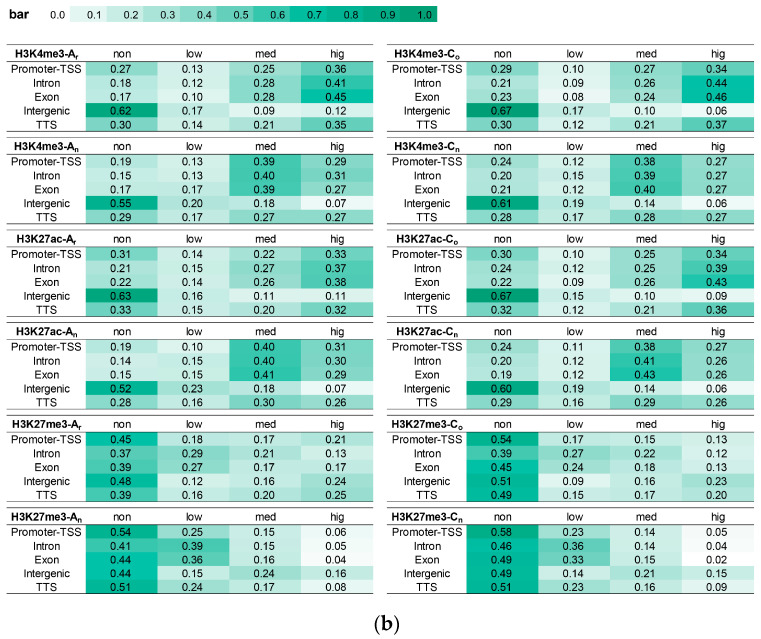
Gene expression characteristics of these genes that were identified as histone modifications in gene regions of *B. napus* and its diploid parents. (**a**) The expression level distribution of the genes whose histone modifications were observed in exons, intergenic regions, introns, and TSS and TTS promoters. (**b**) The proportion of the genes whose exons, intergenic regions, introns, and TSS and TTS promoters were detected as having histone modifications in each expression range.

Histone modification, a key epigenetic mechanism, plays an essential part in controlling gene expression. We explored the impacts of three histone modifications on gene expression in order to discover more about the relationship between gene expression and histone modifications (Figure 11).

The expression levels of genes with H3K27me3 modification in exons, intergenic regions, introns, and TSS and TTS promoter regions were low in *B. napus* and its diploid parents (Figure 11a). The expression levels of H3K4me3- and H3K27ac-modified genes tended to occur at high expression levels, but the expression levels of these genes with intergenic region modifications were low. In terms of proportion (Figure 11b), when the histone modifications H3K4me3 and H3K27ac occurred in the intergenic region, genes closest to the peak center of histone modification tended to have no expression or low expression. Conversely, these genes modified by H3K4me3 and H3K27ac in other gene regions tended to have moderate expression levels and high expression levels. The degree of gene expression tended to be non-expressed or low when H3K27me3 was identified in any gene region.

### 2.8. DNA Methylation Level Variations of DEGs in B. napus

We identified the DEGs between *B. napus* and its diploid parents (Figure S3). Compared to the diploid parents, 2680 DEGs were found in the A_n_ subgenome, and 2302 were found in the C_n_ subgenome; more genes were differentially up-regulated in *B. napus*. DNA methylation is an essential epigenetic mechanism that affects gene expression. According to the results of a correlation study between DNA methylation levels and the gene expression differential multiples in *B. napus* (Figure S4), whether up-regulated or down-regulated genes, the levels of different methylation types in the gene body and promoter regions were positively correlated with the gene expression of differential multiples. Additionally, for all methylation contexts, there was a stronger association between the gene body methylation level and the multiple gene expression differences than there was in promoter regions.

**Figure 12:**
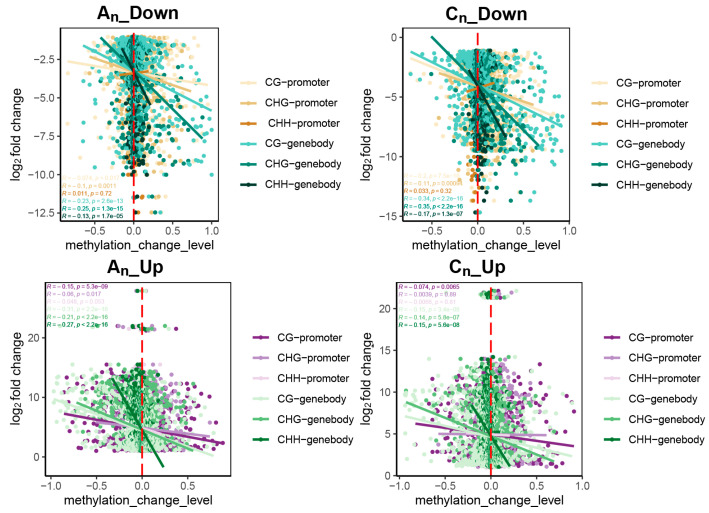
Correlation analysis between DNA methylation level changes and multiple gene expression differences in *B. napus*. The abscissa represents the difference between the methylation levels of the three methylation types in the promoter or gene body of *B. napus* and its diploid parents. A_n__Down and A_n__Up stand for the genes that are down- and up-regulated in A_n_ subgenome, respectively. C_n__Down and C_n__Up represent the C_n_ subgenome’s up- and down-regulated genes, respectively.

Here, we carried out additional research on the association between alterations in DNA methylation levels and variations in gene expression in *B. napus* in reference to its diploid parents (Figure 12).

The methylation levels in the promoter and gene body regions of DEGs both increased and decreased. Furthermore, it is evident from the distribution of points in the figure that the majority of the DEGs in *B. napus* had minor methylation alterations, with most of these changes being around 0. However, for genes that were down-regulated, the higher the increase in DNA methylation level, the bigger the differential down-regulated multiple; in contrast, for genes that were up-regulated, the greater the reduction in DNA methylation level, the bigger the differential up-regulated multiple. This correlation was greater in the gene body than in promoter regions.

According to these results, the methylation levels of gene body and promoter regions and the differential multiples of DEGs in allopolyploid *B. napus* were positively correlated. The degree of variation in DNA methylation levels also had a consequence on the differential multiples of these DEGs.

### 2.9. Combined Consequences of DNA Methylation and Histone Modification on Gene Expression

**Figure 13:**
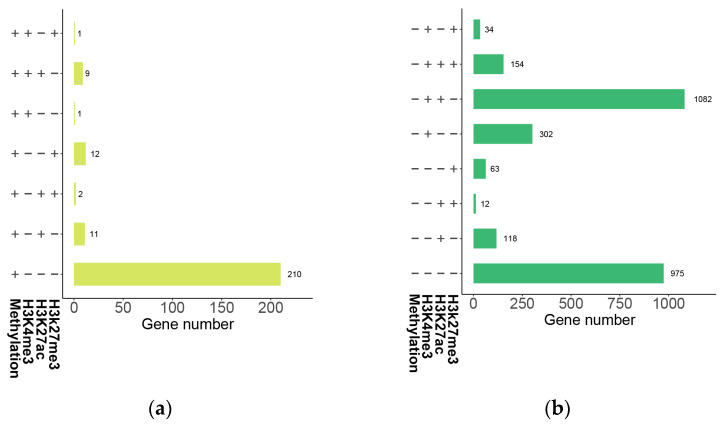
Histone modifications of the genes with highly negative correlations between gene expression and CG methylation levels in the gene body and promoter in *B. napus*. (**a**) Histone modifications of the genes that are fully methylated but not expressed. (**b**) Histone modifications of the genes with non-methylation but high expression.

Based on the analysis of the effects of DNA methylation on gene expression described above (Figure 8a), a total of 2986 genes of *B. napus* were identified with conservative CG methylation levels and a strongly negative correlation with gene expression. Because there are only a few (less than 10) methylated CHG and CHH genes in this study, only the genes with fully methylated CG levels and no expression were considered. Then, we carried out an analysis of the histone modifications of those 2986 genes (Figure 13).

The great majority (210 genes, roughly 85%) of unexpressed genes had full CG methylation, but no H3K4me3, H3K27ac, or H3K27me3 histone modifications were identified. Approximately 36% (975 genes) of the highly expressed genes maintained extremely low CG methylation levels (methylation level less than 0.1), and no H3K4me3, H3K27ac, or H3K27me3 histone modifications were found. For 1082 high expression genes (about 39%) with unmethylated CG, H3K4me3 and H3K27ac histone modifications were detected, but H3K27me3 histone modifications were not discovered. As a result, high CG methylation levels can inhibit gene expression without cooccurring with H3K27me3 modifications. However, for non-methylated and high-expression genes, genes with both H3K4me3 and H3K27ac modifications are more likely to be highly expressed than those that are non-methylated and have no histone modifications.

**Figure 14:**
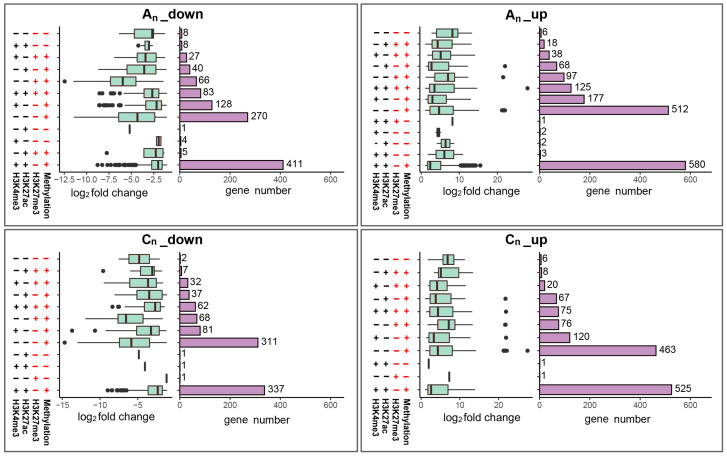
The consequences of DNA methylation and histone modification on the DEGs in *B. napus*. Here, “+” represents an occurrence of corresponding epigenetic modification, whereas “–” denotes the absence of corresponding epigenetic modification. The distribution of log_2_ fold change values for each category of DEGs is displayed in a boxplot. The number of DEGs in each category is depicted in the bar graph.

The two subgenomes of *B. napus* had a total of 4982 DEGs in comparison to its diploid parents. The factors influencing gene expression are complicated, and there is a correlation between DNA methylation or histone modification with differential gene expression. The implications of DNA methylation, H3K4me3, H3K27ac, and H3K27me3 modifications on the DEGs in *B. napus* were, therefore, thoroughly explored (Figure 14).

According to the analysis, 4934 (about 99%) DEGs were discovered with DNA methylation, and 1556 (about 31%) DEGs only showed DNA methylation. Additionally, 1853 (37%) DEGs exhibited DNA methylation and H3K4me3 and H2K27ac modifications in the two subgenomes. In both subgenomes, the number of up-regulated DEGs that displayed only DNA methylation was more than that of down-regulated DEGs, which may be connected to the position of DNA methylation.

For histone modification that promoted gene expression, only H3K4me3 and H3K27ac were modified in the 13 DEGs that were detected. For histone modification that inhibited expression, only H3K27me3 was detected in two DEGs. Furthermore, about 99% of DEGs were detected with DNA methylation, while a small number of DEGs were discovered with no DNA methylation or just histone modification, demonstrating that gene expression differences in *B. napus* were closely linked to DNA methylation. At least two or more modifications of DNA methylation, H3K4me3, H3K27ac, and H3K27me3, were identified in 3392 DEGs (about 68%), suggesting that genes differentially expressed in *B. napus* were regulated by multiple modifications.

The above results demonstrate that DNA methylation is crucial for differential gene expression in *B. napus,* and most DEGs require at least two modifications to regulate the process.

## 3. Discussion

### 3.1. Gene Expression Alteration and Asymmetric Subgenome Epigenetic Modification in B. napus

The expression of genes during plant growth, development, and stress response is significantly influenced by DNA methylation [15,27,28,29]. The A_n_ and C_n_ subgenomes exhibit different distributions of epigenetic modifications, with the former having a higher level of active epigenetic markers and a lower level of inhibitory epigenetic modifications [26]. Biased epigenetic modifications in allopolyploids are involved in regulating the formation of subgenomic dominance [30]. The present results demonstrated that genes in diploid parents with no and high expression levels tended to shift to low and medium expression levels in *B. napus*. The C_n_ subgenome of *B. napus* exhibited higher alterations in gene expression compared to its diploid parents, which may be caused by the following two reasons. First, the C_n_ subgenome is larger than the A_n_ subgenome. This disparity in gene number due to genome size may result in more genes in the C_n_ subgenome having various expression levels. Additionally, asymmetric epigenetic modification may lead to differences in expression levels between subgenomes. Most genes are expressed at lower levels in *B. napus* compared to its diploid parents. Meanwhile, we identified more DEGs in the A_n_ subgenome, while the number of differently up-expressed genes exceeded the number of differently down-expressed genes. Both cotton [31] and *B. napus* [32] have been demonstrated to have the expression level bias of homologous genes. Subgenomic dominance, which may be associated with the biased loss and separation of genes, is shown when the subgenomic expression level is biased [20].

In the A_n_ and C_n_ subgenomes, more genes were identified as having increased methylation levels in promoter and gene body regions. This may be one of the reasons for the decreased gene expression in allopolyploid *B. napus*. Histone modification results revealed that the C_n_ subgenome of *B. napus* had a larger peak number of H3K4me3, H3K27ac, and H3K27me3 modifications than the A_n_ subgenome, suggesting that the C_n_ subgenome had more histone modifications. An analysis of histone modification changes showed that the C_n_ subgenome identified more conserved histone modification than the A_n_ subgenome. Subgenome-biased gene expression and epigenetic modification are important features of allopolyploids, and subgenome-biased H3K4me3 modification has been observed in allotetraploid cotton [11].

### 3.2. Various Epigenetic Markers Are Involved in Regulating Gene Expression in B. napus

DNA methylation is an essential epigenetic modification that regulates gene expression and can be influenced by genetic and epigenetic modifications [33]. DNA hypomethylation was discovered to inhibit hundreds of genes during tomato ripening [34]. In addition, DNA methylation may also promote gene expression; for example, SUVH1 DNA methylation in promoter regions promoted the expression of some genes in Arabidopsis [35]. The results of our study revealed that the degree of variation in DNA methylation levels also had an impact on the differential expression multiples of these DEGs, which were positively correlated with the methylation levels of the gene body and promoter.

The maintenance of DNA methylation is intimately tied to histone modification. One type of histone modification can promote the addition of the same type of further modification and the removal of the opposite type of modification [36]. Histone modification is involved in regulating differential gene expression in rice under salt stress [37]. In allotetraploid cotton leaf tissue, H3K4me3 and H3K27me3 had both direct and indirect impacts on the differential transcription of genes or homologous gene pairs between subgenomes [38]. The expression of low-temperature-stress-related genes can be regulated by the antagonistic relationship between H3K27ac and H3K27me3 in rice [39]. Epigenetic marks usually regulate gene expression synergistically rather than independently, and some DNA methylases can directly interact with histone-modifying enzymes [40]. Nearly all DEGs were identified to be methylated in the present study, and more than half of these genes had more than two epigenetic modifications. Such results imply that DNA methylation is crucial for the formation of DEGs in *B. napus*. Various epigenetic modifications are involved in regulating the creation of DEGs in *B. napus* rather than being regulated by a single epigenetic modification. Histone acetylation also co-regulates gene transcription with other epigenetic modifications [41]. There is a self-reinforcing loop between histone modification and DNA methylation [42]. An interaction occurred between H3K9 methylation and DNA methylation [43]. H3K4me3 is associated with gene expression, but H3K4me1 or H3K4me2 may not be directly involved in transcriptional activation, and H3K4me2/3 modification and DNA methylation appear to be mutually exclusive [44]. Inhibitory H3K27me3 is often enriched with active markers (H3K4me3 and/or H3K27ac) that jointly label enhancers [45]. These studies indicate that the regulation of gene expression by DNA methylation or histone modification involves complex regulatory networks.

## 4. Materials and Methods

### 4.1. Plant Materials

Resynthesized tetraploid *B. napus* cv. ‘HC-2’ (2n = 4x = 38, AACC) and its diploid parents *B. rapa* cv. ‘9JC002’ (2n = 20, AA) and *B. oleracea* cv. ‘3YS013’ (2n = 18, CC) were used as sequencing materials. The seeds were provided by the Oil Crops Research Institute, Chinese Academy of Agricultural Sciences (Wuhan, China). All plant growth and treatment occurred in an incubator (model: HP250G-C, 22 °C, day/night: 16 h/8 h). The plants were planted in vermiculite and irrigated with 1/2 Hoagland nutrient solution (Ph = 5.8) for 30 days to ensure they grew normally. Three plants from each species with healthy growing conditions and consistent growth were selected as sequencing materials.

### 4.2. Transcriptome Sequencing and RT-qPCR

Transcriptome sequencing was completed using the Illumina (San Diego, CA, USA) NovaSeq 6000 sequencing platform with a sequencing mode of 150PE, and each sample was not less than 6 Gb of data. The low-quality data of the sequencing data were intercepted by Trimmomatic software [46], and clean data were obtained. Taking the tetraploid *B. napus* genome (Bna 4.1) as a reference genome, high-quality reads of *B. napus* and its diploid parents were aligned to the genome by the comparison software HISAT [47] (hierarchical indexing for spliced alignment of transcripts).

RNA was extracted and reverse-transcribed into cDNA. Then, 9 genes were randomly selected to verify gene expression. Primer 5 was used for primer design, and the primer sequence is shown in Table S1.

### 4.3. Analysis of Distribution Characteristics of Gene Expression

The HTseq package was used for alignment (intersection_nonempty method) [48]. Gene expression was standardized by the fragments per kilobase per million (FPKM) method. According to the FPKM values, gene expression levels were divided into the following four intervals: less than 0.001 for no expression, greater than or equal to 0.001 for low expression, greater than or equal to 5 for medium expression, and greater than or equal to 50 for high expression. The ggplot2 tool [49] was used to display the number and proportion of genes in each expression level interval, and TBtools [50] was used to analyze the changes of gene expression levels in *B. napus* and its diploid parents and plot upset maps. Information regarding the gene duplication types seen in *B. napus* was obtained from PlantDGD (http://pdgd.njau.edu.cn:8080, accessed on 9 March 2021) [51].

### 4.4. Screening of Differential Expression Genes (DEGs) and Annotation

DEGs were detected by DESeq2 [52]. A log_2_ fold change ≥ 1 and *p*-value ≤ 0.001 were considered to indicate up-regulated expression. If the log_2_ fold change was ≤ −1 and *p* value was ≤ 0.001, gene expression was considered down-regulated. TBtools were used for the GO annotation and the KEGG pathway analysis of DEGs [50], and ggplot2 was used to display annotation terms [49].

### 4.5. Whole-Genome Bisulfite Sequencing (WGBS)

Whole-genome bisulfite sequencing (WGBS) libraries were constructed, and the sequencing strategy was MGI PE150 sequencing. Bsmap software [53] was used to align methylation data to the reference genome. For the methylation site, the methylation level was calculated as follows: ML = mC/(mC + umC) (where ML is the methylation level). The methylation level of each methylated C base was calculated as follows: methylation rate at the C site = 100 × Supporting methylation reads/(supporting methylated reads + supporting non-methylated reads). The proportion of methylated cytosine was calculated as follows: the mC in each context/the number of C in each context (e.g., mCG proportion = mCG/CG).

### 4.6. DNA Methylation Level Analysis

Regarding the average methylation level of the whole genome, the methylation levels of each methylated C site were calculated according to the following formula: methylation rate at site C = 100 × reads/(reads that support methylation + reads that support non-methylation). The genes were divided into promoters (2000 bp upstream of the TSS) and gene body regions, and the methylation level was statistically analyzed. Methylation levels were divided into unmethylated (non-methylated), low (low methylation), moderate (moderate methylation), hig (high methylation) and methylated (complete methylation) across five intervals, namely, [0, 0.1), [0.1, 0.4), [0.4, 0.6), [0.6, 0.9), and [0.9, 1]. Based on the information on the gene duplication types in *B. napus*, the methylation levels of different duplication genes in the promoter and gene body regions of *B. napus* and its diploid parents were analyzed.

### 4.7. Histone Modification Analysis

The histone modification information of *B. napus* and its diploid parents was detected by chromatin immunoprecipitation and sequencing (ChIP-seq) technology [54]. The distribution of histone modification levels in all gene regions was analyzed through the pileup value. Histone modification information was combined with gene duplication types to analyze the relationship between histone modification and duplication genes.

## 5. Conclusions

In conclusion, this study analyzed the features of subgenomic gene expression, DNA methylation, and histone modifications and explored the influence of epigenetic modifications on the regulation of gene differential expression in *B. napus*. The genes in diploid parents that exhibited high and no expression tended to exhibit medium and low expression levels in *B. napus*. The DNA methylation level of *B. napus* increased in comparison to the diploid parents, as did the peak number of H3K4me3 modifications, while H3K27me3 and H3K27ac modifications reduced. These results provide a reference for understanding the molecular mechanism of epigenetic regulation of allopolyploids.

## Figures and Tables

**Table 1:** The peak number of histone modifications in *B. napus* and its diploid parents.

Class	A_r_	A_n_	C_o_	C_n_
H3K4me3	16,902	21,674	24,070	24,396
H3K27ac	21,249	18,417	25,959	21,808
H3K27me3	11,393	10,118	15,303	11,656

## Data Availability

All data analyzed during this study are included in this article and Supplementary Materials. The raw data have been uploaded to NCBI Sequence Read Archive (SRA) database with accession numbers SRR21289120-SRR21289126 and SRR21289135-SRR21289136 (RNA-seq); SRR21383417-SRR213834123 and SRR21383432-SRR21383433 (WGBS); SRR13318007-SRR13318013, SRR13318015, SRR13318018-SRR13318022, and SRR13318025-SRR13318030 (ChIP-seq).

## Supplementary Materials

The following supporting information can be downloaded at: https://www.mdpi.com/article/10.3390/plants12142608/s1, Figure S1: Proportion of genes for different DNA methylation levels in promoter regions of *B. napus* and its diploid parents in different expression levels. The darker the blue, the higher the gene number proportion in the promoter and gene body regions. The title of the first row of the first column of each heat map represents three methylation types in the promoter region of the two subgenomes of *B. napus* and its diploid parent, e.g., promoter_CG_A_r_ represents CG methylation in the promoter region of *B. rapa*, and so on; Figure S2: Proportion of genes at different DNA methylation levels in gene body regions of *B. napus* and its diploid parents in different expression levels. The darker the green, the higher the gene number proportion in the promoter and gene body regions. The title of the first row of the first column of each heat map represents three methylation types in the gene body region of the two subgenomes of *B. napus* and its diploid parent, e.g., gene body_CG_A_r_ represents CG methylation in the gene body region of *B. rapa*, and so on; Figure S3: Volcano map of differential expression genes of *B. napus* compared with its diploid parents. A_r__A_n_ represents the comparison of the *B. napus* A_n_ subgenome gene with *B. rapa*, and C_o__C_n_ indicates the comparison of the *B. napus* C_n_ subgenome gene with *B. oleracea*. The terms Down, Not Change, and Up denoted down-regulated, no difference in expression level, and up-regulated expression genes in comparison to diploid parents in *B. napus*, respectively; Figure S4: Analysis of the correlation between DNA methylation levels and gene expression of differential multiples in *B. napus*. The abscissa represents the methylation levels of the three methylation types of the promoter or gene body. A_n__Down and A_n__Up stand for the genes that are down- and up-regulated in A_n_ subgenome, respectively. C_n__Down and C_n__Up represent the C_n_ subgenome’s up- and down-regulated genes, respectively. Table S1. Sequences of gene primers involved in RT-qPCR experiment.
