# Supplementary figures and images for: Epigenetic Regulation of Subgenomic Gene Expression in Allotetraploid Brassica napus
Source: Plants (Basel). 2023 Jul 10;12(14):2608. doi: 10.3390/plants12142608 (PMC10383903; doi:10.3390/plants12142608)

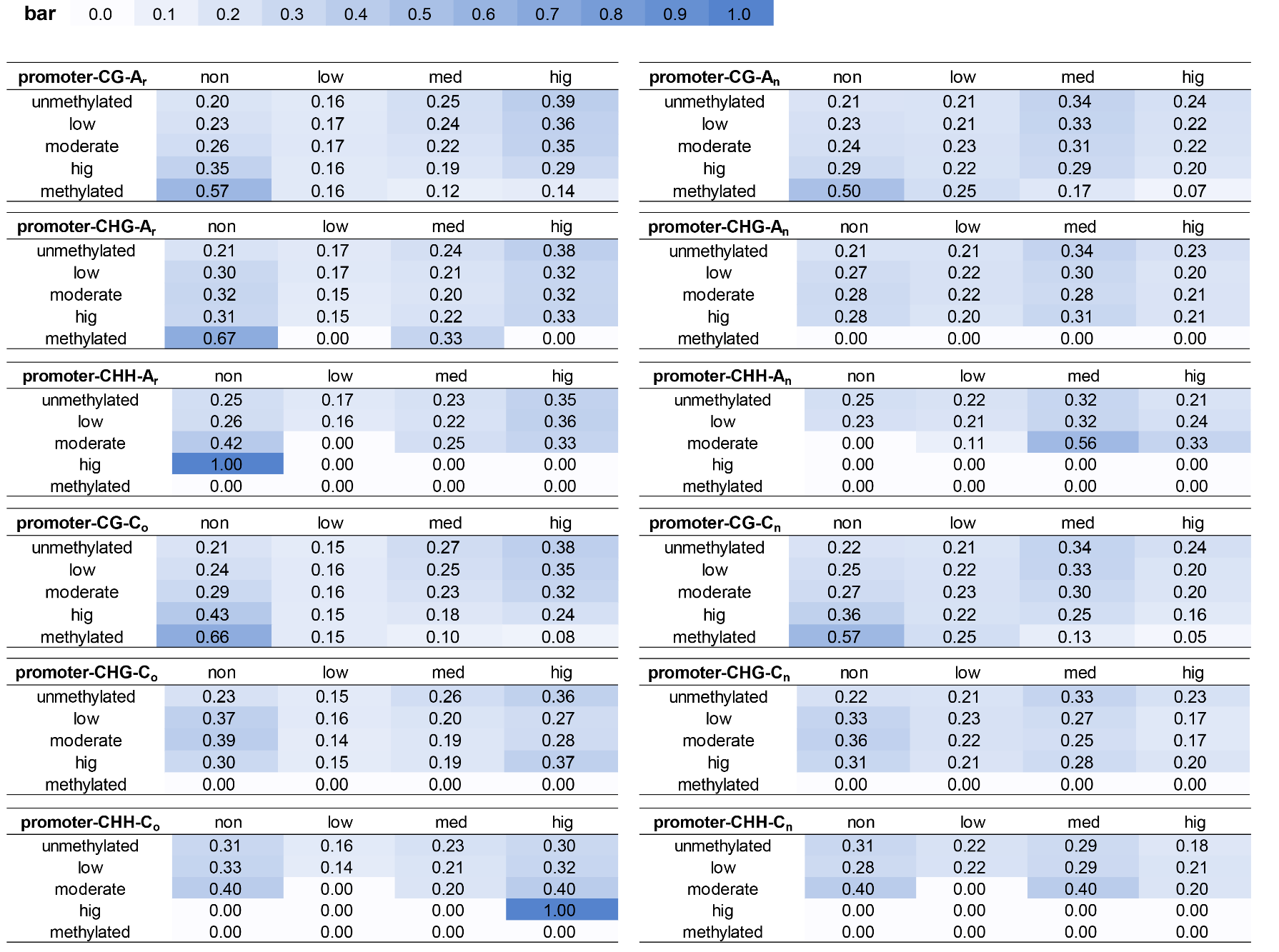

Supplement: Supplementary file 1 [file plants-12-02608-s001.zip › Figure S1.tif]

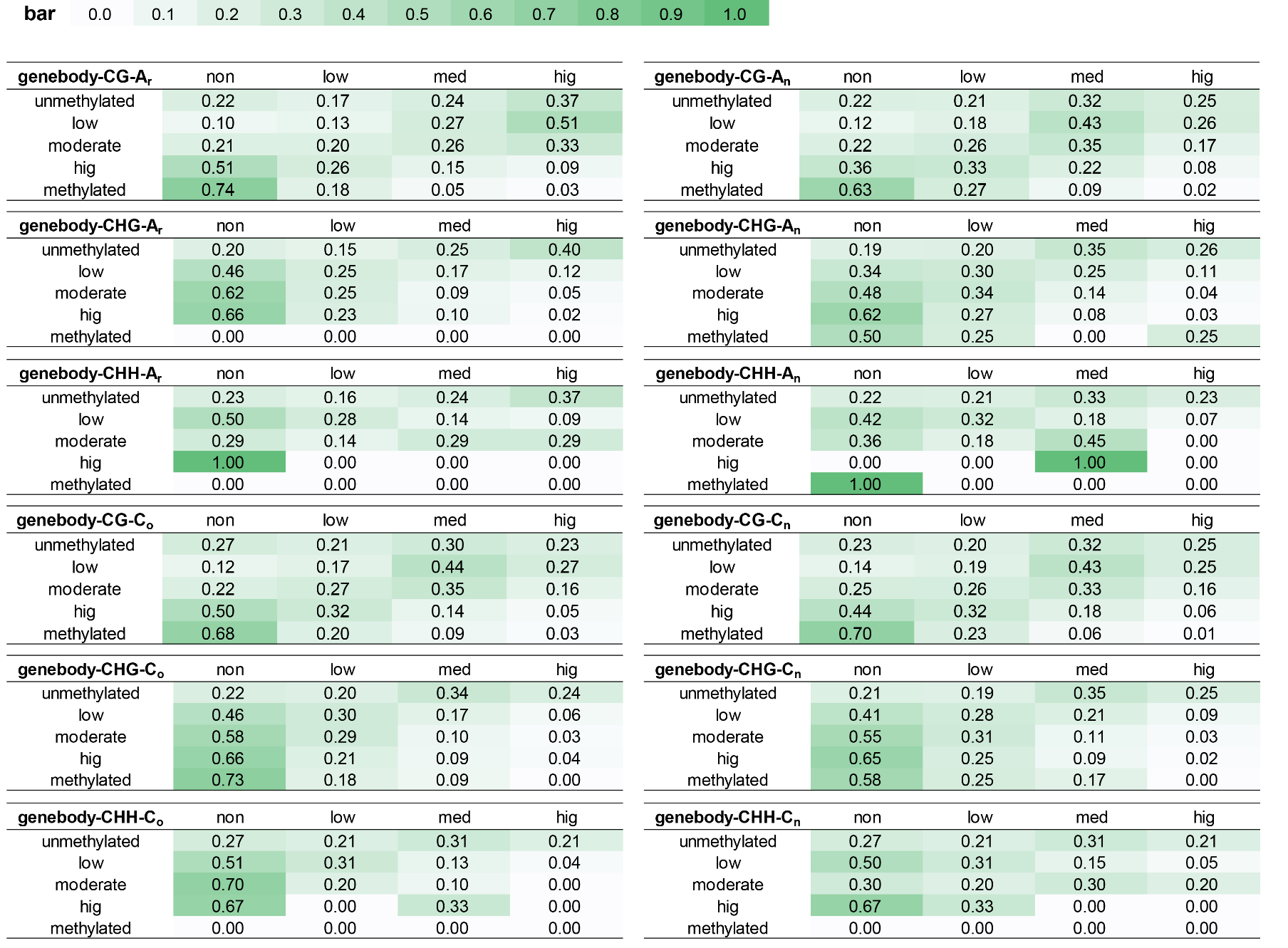

Supplement: Supplementary file 1 [file plants-12-02608-s001.zip › Figure S2.tif]

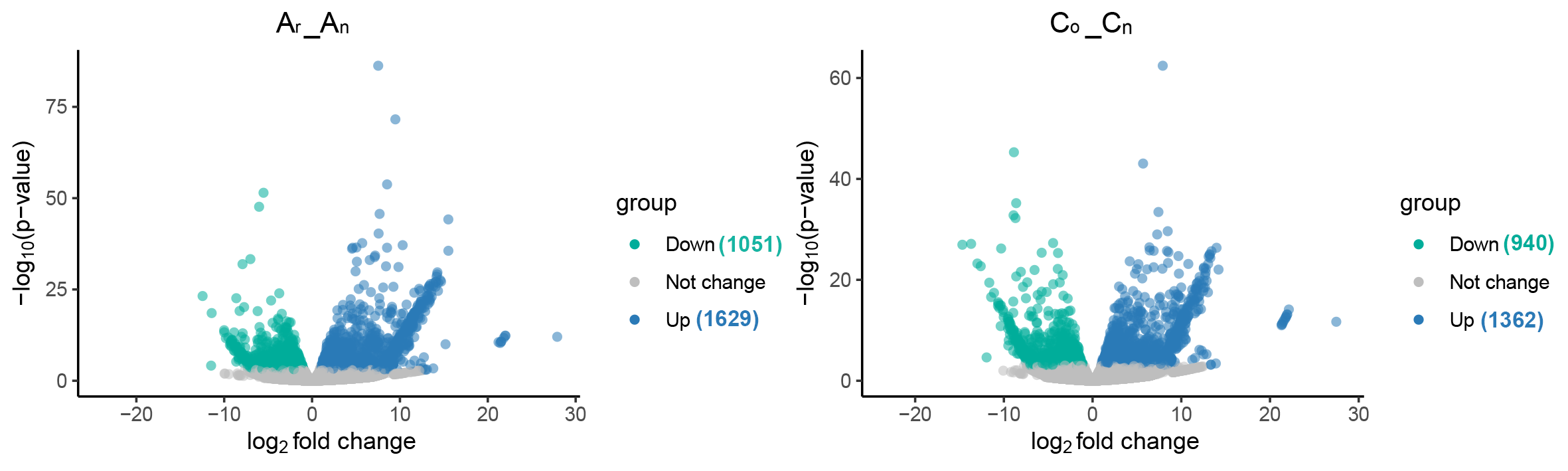

Supplement: Supplementary file 1 [file plants-12-02608-s001.zip › Figure S3.tif]

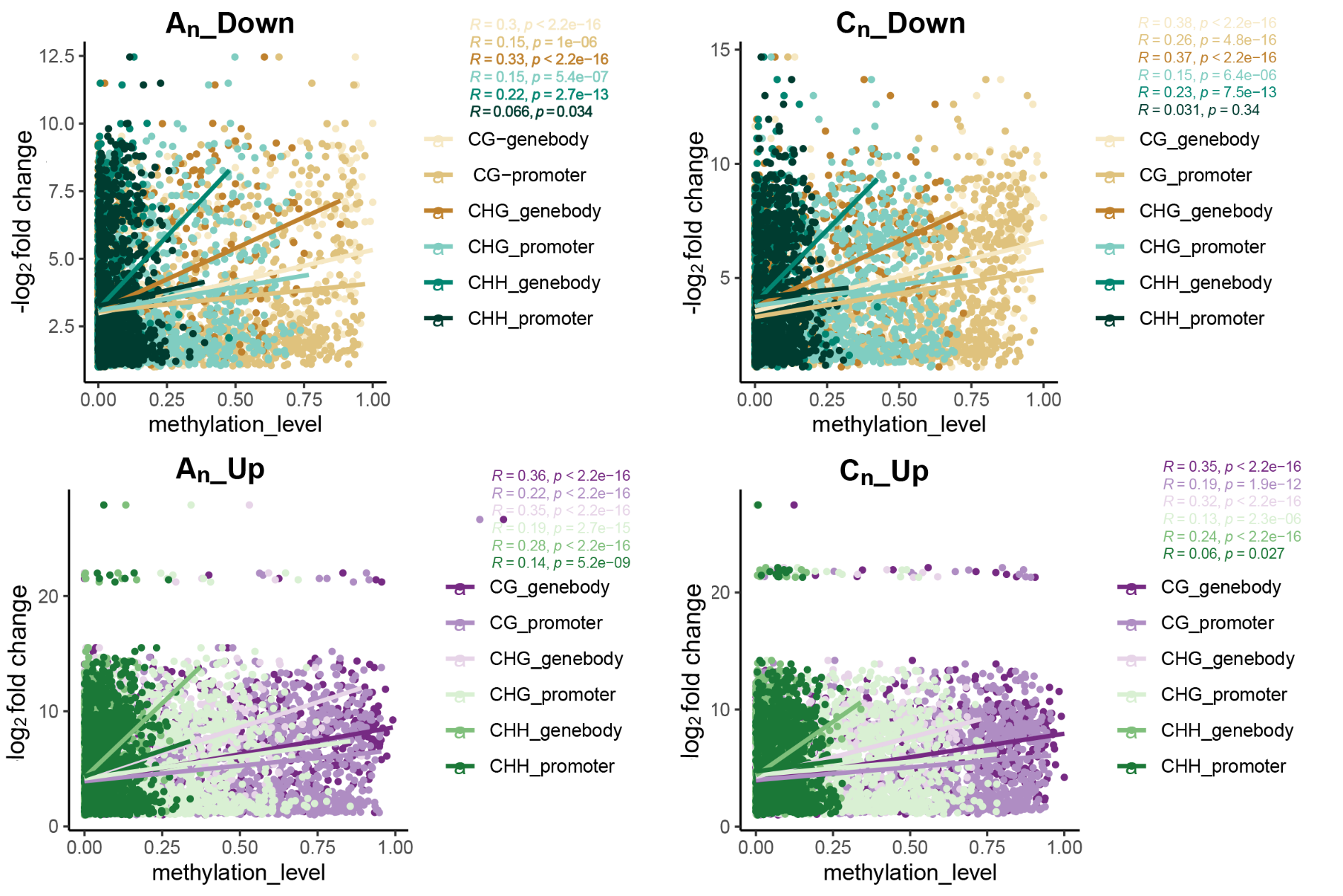

Supplement: Supplementary file 1 [file plants-12-02608-s001.zip › Figure S4.tif]
